# A repeated cross-sectional analysis of SARS-CoV-2 seroprevalence in Manila, the Philippines after implementation of the national COVID-19 vaccination program

**DOI:** 10.1186/s41182-025-00767-9

**Published:** 2025-06-16

**Authors:** Greco Mark B. Malijan, Shuichi Suzuki, Ana Ria Sayo, Annavi Marie Villanueva, Kristal An Agrupis, Abigail Ortal-Cruz, Mary Ann Salazar, Jan Wendzl Evangelista, Rontgene Solante, Grace Devota Go, Naomi Ruth Saludar, Dinarazad Miranda, Alexis Dimapilis, Koya Ariyoshi, Chris Smith

**Affiliations:** 1 San Lazaro Hospital – Nagasaki University Collaborative Research Office, San Lazaro Hospital, Quiricada Street, Sta. Cruz, 1003 Manila, Philippines; 2 School of Tropical Medicine and Global Health, Nagasaki University, Sakamoto, Nagasaki, 852-8102 Japan; 3 San Lazaro Hospital, Quiricada Street, Sta. Cruz, 1003 Manila, Philippines; 4 Institute of Tropical Medicine, Nagasaki University, Sakamoto, Nagasaki, 852-8523 Japan; 5 Department of Clinical Research, London School of Hygiene & Tropical Medicine, London, WC1E 7HT UK

**Keywords:** COVID-19, Philippines, SARS-CoV-2, Vaccination, Seroepidemiological study

## Abstract

**Background:**

SARS-CoV-2 seroepidemiological studies, which have been used to describe population-level immunity, are limited in the Philippines, despite the protracted course of the epidemic in the country. We follow-up on our previous work and aimed to estimate SARS-CoV-2 seroprevalence and infection rate among outpatient clinic attendees in Metro Manila, a year after the implementation of the national COVID-19 vaccination program.

**Methods:**

We conducted four repeated cross-sectional surveys at the outpatient department of San Lazaro Hospital between March 2022 and January 2023. We performed χ^2^ test and analysis of variance to assess the differences in characteristics across different data collection periods.

**Results:**

A total of 765 participants were enrolled, ranging from 170 to 200 per period. Participant demographic, socioeconomic, and medical history were comparable across all data collection periods. Between March and October 2022, the proportion of participants who received a vaccine or booster dose significantly increased, from 77.9% to 90%. Seroprevalence across all data collection periods was consistently high, ranging from 97.8% to 99.5%. However, the geometric mean concentration of antibodies was highest in the data collection period following the Omicron-dominant wave. Infection rates were comparably low (< 10%) across periods, except for a peak at 16.7% in September to October 2022, which followed the rise in reported cases in Metro Manila.

**Conclusion:**

Population-level seroprevalence among clinic attendees in Manila was consistently high a year after implementation of the national COVID-19 vaccination program, but analyses of antibody concentrations showed potential waning within a 3-month period.

## Background

Severe acute respiratory syndrome coronavirus 2 (SARS-CoV-2)-specific humoral and cellular immune responses are critical to suppressing viral replication, reducing severity of illness, and preventing reinfection [1, 2]. Evaluation of the effect of allocation to neutralizing monoclonal antibodies compared to placebo showed that active treatment accelerated the natural decline in viral load over time and reduced coronavirus disease 2019 (COVID-19)-related hospitalization [3]. Similarly, studies on early longitudinal dynamics of T-cell immunity and total and neutralizing antibodies found that upon natural infection, both cellular and humoral responses were coordinated in mild disease but were inconsistent in severe disease [4].

Prior to the availability of vaccines, SARS-CoV-2 seroprevalence studies were conducted to evaluate the true extent of exposure to infection in various populations, according to demographic groups and geographic areas [5, 6]. Seroprevalence rates were used to estimate case underreporting, evaluate the impact of population-level containment measures, parametrize epidemiologic models, and ultimately inform resource allocation [7, 8]. Anchored on the strong relationship between robust humoral immune response and protection from severe disease, seroprevalence studies also aided the evaluation of the average population-level immunity from vaccination programs [5].

Synthesized evidence on the dynamics and durability of antibody response suggest variability according to age, disease severity, history of prior infections, comorbid conditions including immunocompromised status, demographic characteristics, and presence of symptoms [9, 10]. To date, only three seroprevalence studies were conducted in the Philippines, which reported the highest confirmed COVID-19 deaths in the Western Pacific region [11]. We first reported on the seroprevalence in Manila across four periods between May 2020 to March 2021 and found 44.6% seropositivity in the last collection period prior to the national vaccination program [12]. A serial cross-sectional analysis of SARS-CoV-2 antibodies within the community of a private tertiary university in Quezon City conducted in June to December 2021 found 28.8% to 65.1% seropositivity among study population [13]. Finally, a cohort study of confirmed COVID-19 patients between March 2021 to July 2022 explored the durability of antibodies and found that more severe initial infection were associated with higher antibody levels 21 days after initial diagnosis [14].

We conducted repeated cross-sectional surveys to estimate SARS-CoV-2 seroprevalence and infection rate among attendees of an outpatient animal bite clinic, as surrogates for the catchment population, in a tertiary infectious disease referral hospital, in Metro Manila, the Philippines. To provide a more complete estimate of population-level immunity to COVID-19, in this paper we report seroprevalence estimates before and after the implementation of the national COVID-19 immunization program.

## Methods

### Study design

Details about methods of the study and results from Periods 1 to 4 have been previously reported [12, 15]. This repeated cross-sectional analysis is part of the acute respiratory tract infection (ARI) study that aims to describe the epidemiology and clinical features of ARI among patients, healthcare workers, and household contacts in San Lazaro Hospital (SLH) in Metro Manila, the Philippines.

### Setting

Located in the City of Manila, the world’s most densely populated city with more than 43,000 persons/km^2^, SLH—outpatient department animal bite clinic (ABC) provides rabies post-exposure prophylaxis free at point of use for all and receives patients mostly from contiguous cities in Metro Manila. The facility attends to an average of 200 new patients daily.

**Fig. 1:**
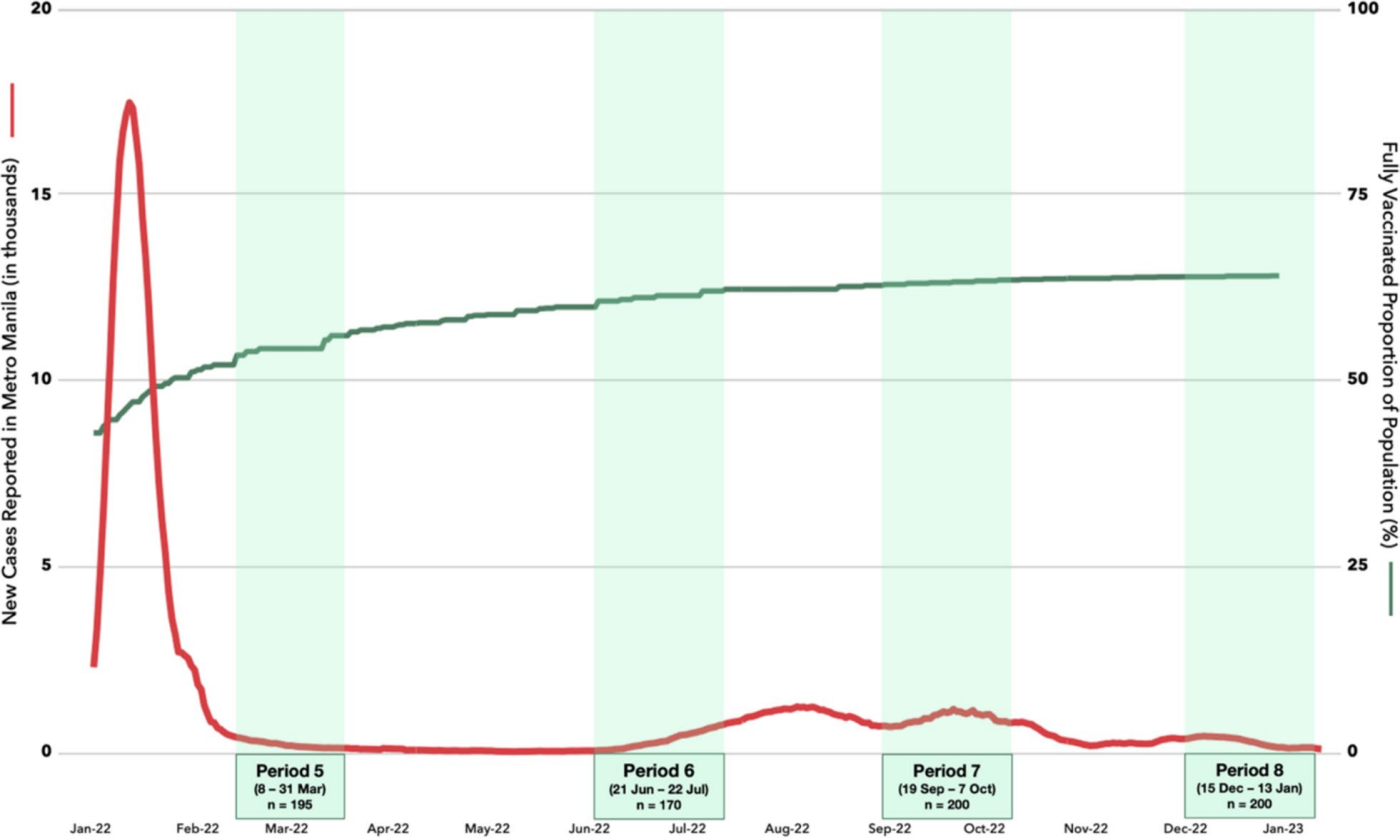
Timing of study enrollment against new cases reported in Metro Manila and national vaccine coverage for primary series among eligible population

Enrollment took place about a year following the implementation of the national COVID-19 vaccination program. Vaccine distribution was primarily coursed through local government units (LGUs) and medical centers. We enrolled patients over four periods, roughly three months apart: 8–31 March 2022 (Period 5), 21 June–22 July 2022 (Period 6), 19 September–7 October 2022 (Period 7), and 15 December 2022–13 January 2023 (Period 8). Period 5 took place immediately after the Omicron-predominant wave. The reported national vaccination coverage for eligible populations reached a plateau by Period 7 (Fig. 1).

### Participants

Patients consulting at ABC and/or their household contacts older than 1 year of age were eligible for enrollment. ABC patients attending for follow-up consult, patients consulting for other medical purposes, and patients triaged to the emergency room were excluded. During each data collection period, trained research nurses systematically approached clinic attendees according to their queue number in the dedicated waiting areas. ABC attendees were invited to participate, and those who provided informed consent were recruited consecutively. There were no limitations on daily recruitment.

### Outcomes

To assess seropositivity, collected serum samples were analyzed using Elecsys Anti-SARS-CoV-2 immunoassay (Roche Diagnostics, Basel, Switzerland) according to manufacturer instructions. The assay uses anti-nucleocapsid (N) antigen and has 99.5% sensitivity and 99.8% specificity within 14 days of infection, based on studies involving symptomatic COVID-19 patients [16, 17]. The cutoff index (COI) for a reactive test is ≥ 1. The test measures total SARS-CoV-2 antibodies including IgG, IgM, and IgA and does not provide immunoglobulin class-specific results [18].

COVID-19 infection was confirmed using real-time reverse transcriptase polymerase chain reaction detecting *RdRP* and *E* genes from extracted viral RNA (Qiagen Viral RNA Mini Kit, Hilden, Germany) from collected nasopharyngeal and oropharyngeal swab specimens using published protocols [19] in the StepOnePlus Real-Time PCR system (Applied Biosystems, Massachusetts, United States).

We operationally defined seroprevalence as the proportion of the population who tested reactive to SARS-CoV-2 antibodies and the infection rate as the proportion of the population with detectable SARS-CoV-2 RNA on RT-PCR.

### Other data

Participant demographics, socioeconomic information, medical history, COVID-19 exposure history, vaccination history, and clinical symptoms were collected via research-assisted questionnaire interview. Data were collected and stored electronically through REDCap [20]. Laboratory results were collated in Microsoft Excel [21] then entered and stored electronically through REDCap.

### Sample size

A minimum sample size of 100 individuals per enrollment period would allow estimation of seroprevalence at least 15% with 10% absolute precision.

### Statistical methods

Participant characteristics were summarized according to data collection period. Continuous data were expressed as mean (SD) and median [Q1, Q3], and categorical data were expressed as number (%). Differences in characteristics across periods were evaluated using Chi-squared test and one-way analysis of variance for categorical and continuous variables, respectively. Seroprevalence and infection rate were reported with 95% binomial confidence intervals. Data cleaning, analysis, and visualization were performed in R version 4.4.2 (R Foundation for Statistical Computing, Vienna, Austria) [22].

### Ethics approval and consent to participate

The study was reviewed and approved by the San Lazaro Hospital—Research Ethics Review Unit (SLH-RERU-2020-022-I) and the Nagasaki University School of Tropical Medicine and Global Health research ethics committee (NU_TMGH_2020_119_1). Informed consent process was conducted in accordance with local regulations and the principles set in the International Conference on Harmonization—Good Clinical Practice.

## Results

### Participants

**Fig. 2:**
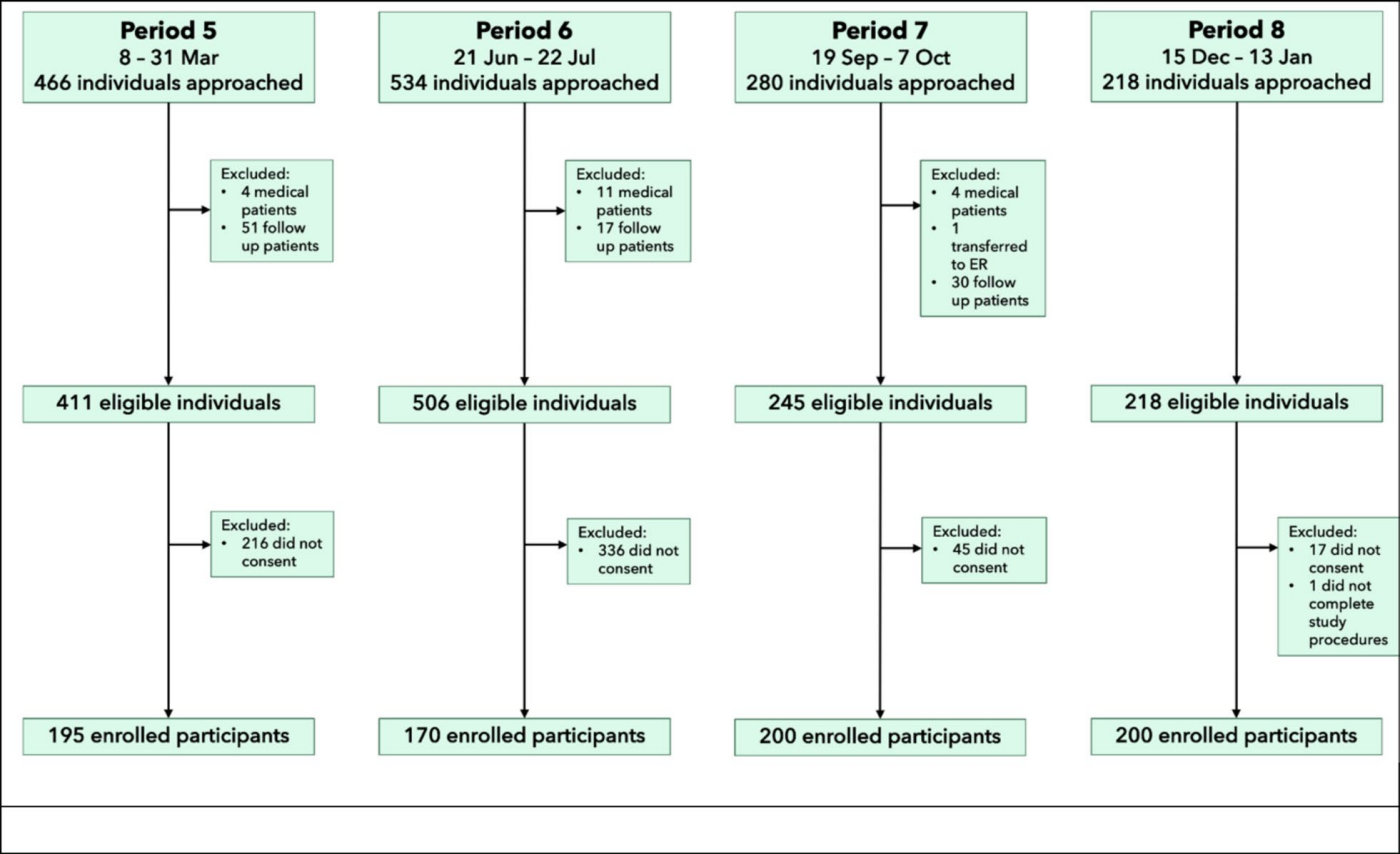
Study participant flow

In total, 765 participants were enrolled, ranging from 170 to 200 per period (Fig. 2). Participant demographic, socioeconomic characteristics, and medical history were comparable across all four data collection periods, except that more participants belonging to economically poorer households (monthly income ≤ 20,000 PHP) were enrolled in Period 5 compared to the later periods and more patients with history of COVID-19 infection were enrolled in Period 6 compared to other periods (Table 1).

**Table 1:** Participant demographics and medical history across periods

	Period 5 8–31 Mar n = 195 (%)	Period 6 21 Jun–22 Jul n = 170 (%)	Period 7 19 Sep–11 Oct n = 200 (%)	Period 8 15 Dec–13 Jan n = 200 (%)	Total N = 765 (%)	p value
Demographics and socioeconomic status						
Sex						
Female	118 (60.5)	108 (63.5)	127 (63.5)	129 (64.5)	482 (63.0)	NS
Age group						
Below 18 years	44 (22.6)	25 (14.7)	23 (11.5)	38 (19.0)	130 (17.0)	NS
18 to 39 years	102 (52.3)	98 (57.6)	103 (51.5)	99 (49.5)	402 (52.5)	
40 to 59 years	38 (19.5)	39 (22.9)	61 (30.5)	54 (27.0)	192 (25.1)	
60 years and above	11 (5.6)	8 (4.7)	13 (6.5)	9 (4.5)	41 (5.4)	
Education						
None	9 (4.6)	2 (1.2)	6 (3.0)	6 (3.0)	23 (3.0)	NS
Primary	39 (20.0)	33 (19.4)	29 (14.5)	38 (19.0)	139 (18.2)	
Secondary	90 (46.2)	81 (47.6)	99 (49.5)	107 (53.5)	377 (49.3)	
Vocational	13 (6.7)	9 (5.3)	14 (7.0)	9 (4.5)	45 (5.9)	
Tertiary	43 (22.1)	43 (25.3)	51 (25.5)	40 (20.0)	177 (23.1)	
Postgraduate	1 (0.5)	2 (1.2)	1 (0.5)	0	4 (0.5)	
Residence						
Metro Manila	182 (93.3)	163 (95.9)	186 (93.0)	190 (95.0)	721 (94.2)	NS
Outside Metro Manila	13 (6.7)	7 (4.1)	14 (7.0)	10 (5.0)	44 (5.8)	
Household size*						
≤ 4 persons	75 (39.3)	63 (37.3)	63 (31.5)	57 (28.5)	258 (33.9)	NS
> 4 persons	116 (60.7)	106 (62.7)	137 (68.5)	143 (71.5)	502 (66.1)	
Monthly household income						
≤ PHP 20,000	164 (84.1)	131 (77.1)	142 (71.0)	136 (68.0)	573 (74.9)	**0.001**
> PHP 20,000	31 (15.9)	39 (22.9)	58 (29.0)	64 (32.0)	192 (25.1)	
Medical history						
Any comorbid illness						
Present	31 (15.9)	41 (24.1)	36 (18.0)	22 (11.0)	108 (19.1)	**0.009**
Frequently reported comorbid illness*						
Hypertension	20 (10.3)	19 (11.2)	22 (11.0)	10 (5.0)	71 (9.3)	NS
History of COVID-19	5 (2.6)	17 (10.0)	11 (5.5)	6 (3.0)	39 (5.1)	**0.005**
Diabetes mellitus	8 (4.1)	4 (2.4)	7 (3.5)	5 (2.5)	24 (3.2)	NS
Bronchial asthma	3 (1.5)	5 (3.0)	1 (0.5)	2 (1.0)	11 (1.4)	NS
History of pulmonary tuberculosis	1 (0.1)	5 (3.0)	1 (0.5)	2 (1.0)	9 (1.2)	NS
Regular smoker*^,a^						
Yes	41 (21.4)	31 (18.5)	40 (20.0)	37 (18.7)	149 (19.7)	NS
Regular alcoholic beverage drinker*^,b^						
Yes	73 (37.8)	69 (40.6)	86 (43.2)	87 (43.5)	315 (41.3)	NS

^*^Missing data, n (%): household size—5 (0.7), hypertension—2 (0.3), diabetes mellitus—4 (0.5), bronchial asthma—6 (0.8), history of pulmonary tuberculosis—8 (1.0), regular smoker—7 (0.9), regular alcoholic beverage drinker—3 (0.4)

^a^Includes individuals who smoke regularly at least a few days a week

^b^Includes individuals who drink regularly at least a few days a week

Bold values indicate p<0.05, NS indicates non-significant

Only 39 of 765 total participants (5.1%) reported having history of COVID-19 infection, 35 of whom reported having only one confirmed episode. Among these participants, the median duration from last COVID-19 illness to study enrollment ranged from 323 days (Period 5) to 533 days (Period 7), without significant difference across periods.

**Fig. 3:**
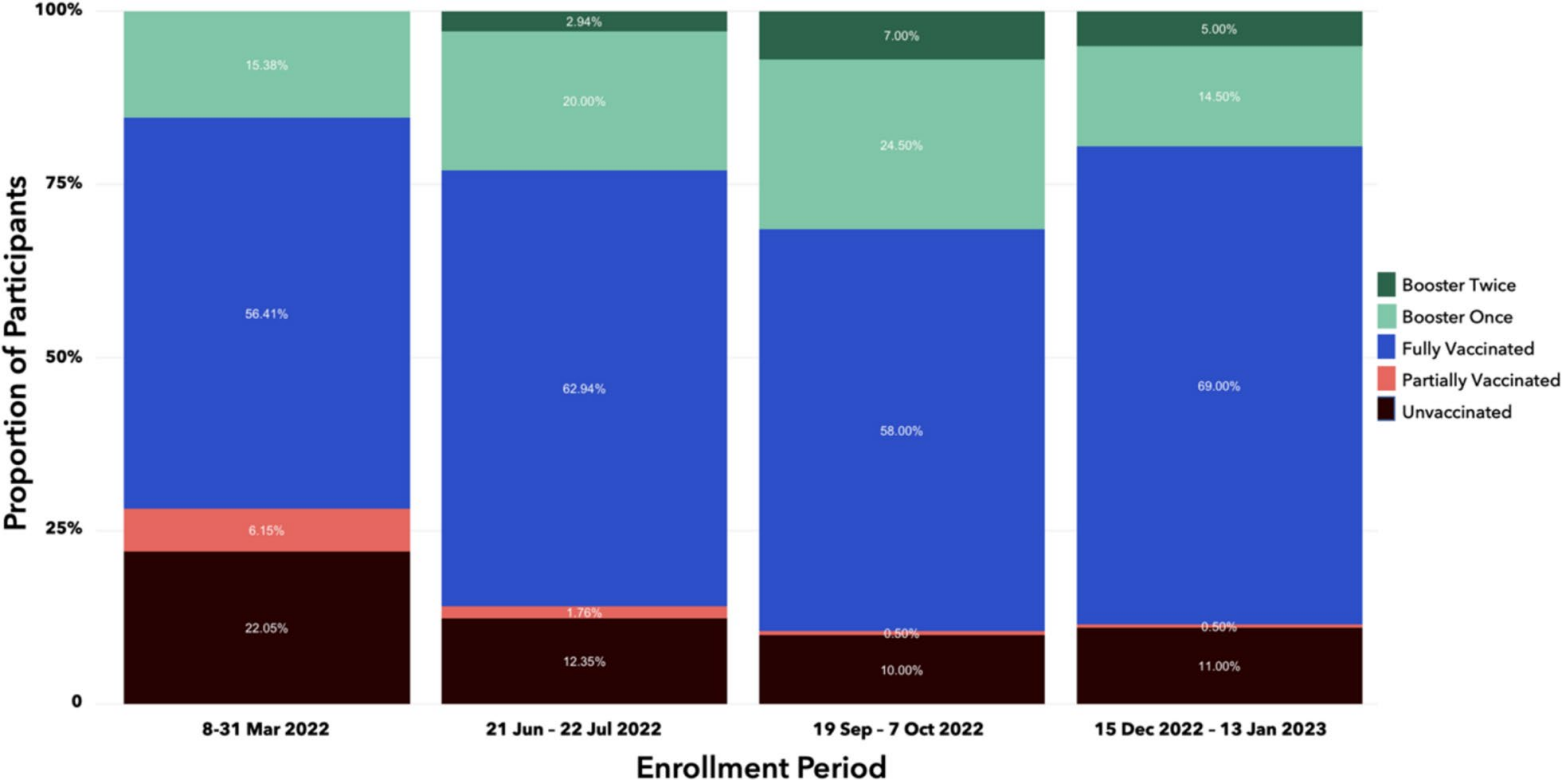
COVID-19 vaccination status of study participants across enrollment periods

Between Period 5 and Period 7 (March and October 2022), the proportion of participants who received full vaccine dose and/or boosters increased significantly and reached a plateau thereafter (Fig. 3), closely mirroring the trend in national vaccination coverage (Fig. 1).

More than 40% of participants received CoronaVac (Sinovac) vaccine as the primary series while majority of those with booster doses received Comirnaty (Pfizer/BioNTech) vaccine (Table 2). There were no significant differences across periods. Among those who received any vaccine, the median duration between last vaccine dose and study enrollment ranged from 113 days (Period 5) to 386 days (Period 8). The majority of the participants received their vaccine doses from either the hospitals or local government units. However, five of 659 (0.8%) who completed primary vaccine series and three of 171 (1.8%) who received any booster dose sourced their immunization through private entities which were not necessarily sanctioned by the government.

**Table 2:** Participant vaccination history

	Period 5 8–31 Mar n = 195 (%)	Period 6 21 Jun–22 Jul n = 170 (%)	Period 7 19 Sep–11 Oct n = 200 (%)	Period 8 15 Dec–13 Jan n = 200 (%)	Total N = 765 (%)	p value
COVID-19 vaccination history						
Vaccination status						
Received booster dose twice	0	5 (2.9)	14 (7.0)	10 (5.0)	29 (3.8)	** < 0.001**
Received booster dose once	30 (15.4)	34 (20.0)	49 (24.5)	29 (14.5)	142 (18.6)	
Received full dose of primary series	110 (56.4)	107 (62.9)	116 (58.0)	138 (69.0)	471 (61.6)	
Received partial dose of primary series	12 (6.2)	3 (1.8)	1 (0.5)	1 (0.5)	17 (2.2)	
Unvaccinated	43 (22.1)	21 (12.4)	20 (10.0)	22 (11.0)	106 (13.9)	
Vaccine brands—first dose						
Comirnaty (Pfizer/BioNTech)	38 (19.5)	37 (21.8)	32 (16.0)	44 (22.0)	151 (19.7)	NS
CoronaVac (Sinovac)	77 (39.5)	71 (41.8)	99 (49.5)	78 (39.0)	325 (42.5)	
Covilo (Sinopharm)	0	1 (0.6)	1 (0.5)	1 (0.5)	3 (0.4)	
Jcovden (Janssen)	5 (2.6)	5 (2.9)	7 (3.5)	6 (3.0)	23 (3.0)	
Spikevax (Moderna)	11 (5.6)	8 (4.7)	22 (11.0)	17 (8.5)	58 (7.6)	
Sputnik V (Gamaleya)	1 (0.5)	0	0	2 (1.0)	3 (0.4)	
Vaxzevria (Oxford/AstraZeneca)	20 (10.3)	26 (15.3)	19 (9.5)	30 (15.0)	95 (12.4)	
Unknown brand	0	1 (0.6)	0	0	1 (0.1)	
No vaccine	43 (22.1)	21 (12.4)	20 (10.0)	22 (11.0)	106 (13.9)	
Vaccine brands—second dose						
Comirnaty (Pfizer/BioNTech)	32 (16.4)	38 (22.4)	31 (15.5)	43 (21.5)	144 (18.8)	NS
CoronaVac (Sinovac)	74 (37.9)	69 (40.6)	98 (49.0)	77 (38.5)	318 (41.6)	
Covilo (Sinopharm)	0	1 (0.6)	1 (0.5)	1 (0.5)	3 (0.4)	
Spikevax (Moderna)	11 (5.6)	8 (4.7)	22 (11.0)	17 (8.5)	58 (7.6)	
Sputnik V (Gamaleya)	1 (0.5)	0	0	2 (1.0)	3 (0.4)	
Vaxzevria (Oxford/AstraZeneca)	20 (10.3)	24 (14.1)	19 (9.5)	30 (15.0)	93 (12.2)	
Unknown brand	0	1 (0.6)	1 (0.5)	1 (0.5)	3 (0.4)	
No vaccine	60 (30.8)	29 (17.1)	28 (14.0)	29 (14.5)	146 (19.1)	
Vaccine brands—first booster						
Comirnaty (Pfizer/BioNTech)	15 (7.7)	18 (10.6)	33 (16.5)	26 (13.0)	92 (12.0)	NS
CoronaVac (Sinovac)	2 (1.0)	2 (1.2)	9 (4.5)	4 (2.0)	17 (2.2)	
Spikevax (Moderna)	0	10 (5.9)	7 (3.5)	2 (1.0)	19 (2.5)	
Vaxzevria (Oxford/AstraZeneca)	5 (2.6)	8 (4.7)	14 (7.0)	7 (3.5)	34 (4.4)	
Unknown brand	8 (4.1)	1 (0.6)	0	0	9 (1.2)	
No booster	165 (84.6)	131 (77.1)	137 (68.5)	161 (80.5)	594 (77.6)	
Vaccine brands—second booster						
Comirnaty (Pfizer/BioNTech)	0	3 (1.8)	13 (6.5)	8 (4.0)	24 (3.1)	NS
CoronaVac (Sinovac)	0	0	1 (0.5)	0	1 (0.1)	
Spikevax (Moderna)	0	2 (1.2)	0	1 (0.5)	3 (0.4)	
Vaxzevria (Oxford/AstraZeneca)	0	0	0	1 (0.5)	1 (0.1)	
No booster	195 (100.0)	165 (97.1)	186 (93.0)	190 (95.0)	736 (96.2)	
Duration between last vaccine dose and data collection (days)						
Median [IQR]	113 [53, 188]	219 [147, 290]	281 [225, 385]	386 [308, 483]	250 [144, 364]	** < 0.001**
Primary series vaccine source	n = 152	n = 149	n = 180	n = 178	N = 659	
Hospitals	2 (1.3)	3 (2.0)	6 (3.3)	3 (1.7)	14 (2.1)	NS
Local government units	144 (94.7)	145 (97.3)	172 (95.6)	173 (97.2)	634 (96.2)	
Private agencies	1 (0.7)	1 (0.7)	1 (0.5)	2 (1.1)	5 (0.8)	
Others	5 (3.3)	0	1 (0.6)	0	6 (0.9)	
Booster dose source	n = 30	n = 39	n = 63	n = 39	N = 171	
Hospitals	1 (3.3)	2 (5.1)	6 (9.5)	4 (10.3)	13 (7.6)	NS
Local government units	27 (90.0)	35 (98.7)	56 (88.9)	34 (87.2)	152 (89.4)	
Private agencies	1 (3.3)	1 (2.6)	0	1 (2.6)	3 (1.8)	
Others	1 (3.3)	1 (2.6)	1 (1.6)	0	3 (1.8)	
Other vaccines						
Influenza vaccine*	12 (6.2)	18 (10.6)	12 (6.0)	22 (11.0)	64 (8.4)	NS
Pneumococcal vaccine*	7 (3.6)	4 (2.4)	10 (5.0)	8 (4.0)	29 (3.8)	NS
Diphtheria/pertussis-containing vaccine*	28 (14.7)	17 (10.2)	69 (34.5)	110 (55.0)	224 (29.6)	** < 0.001**
Bacille Calmette–Guerin vaccine*	156 (81.2)	150 (89.3)	162 (81.4)	198 (100.0)	666 (88.0)	** < 0.001**

^*^Missing data, n (%): influenza vaccine—1 (0.1), pneumococcal vaccine—5 (0.7), diphtheria/pertussis-containing vaccine—8 (1.0), Bacille Calmette–Guerin vaccine—8 (1.0)

Regarding the prevalence of immunization for other respiratory infections, < 10% of participants received influenza vaccine in the past year and < 5% received pneumococcal vaccine in the past 5 years, with no significant difference across periods. The proportion of participants vaccinated against diphtheria and/or pertussis in the past 10 years was almost four times greater in Period 8 (55.0%) compared to Period 5 (14.7%). Finally, the prevalence of BCG vaccination history was high across periods, with all of participants ever receiving the vaccine to protect against tuberculosis during the last enrollment period.

### Seroprevalence

Across all data collection periods after the implementation of the national COVID-19 vaccination program, the seroprevalence was consistently high, ranging from 97.8% to 99.5% (Table 3). However, the geometric mean concentration (GMC) of antibodies among seropositive individuals was highest in Period 5 (128 COI), which immediately followed the Omicron-dominant wave. The GMC in Period 6, which was conducted about three months from Period 5, was almost half of the earlier measurement (54.2 COI). Finally, there was a comparable but increasing trend in GMC in the succeeding periods, with 66.9 COI in Period 8.

**Table 3:** Infection rate and seroprevalence

	Period 5 8–31 Mar n = 195 (%)	Period 6 21 Jun–22 Jul n = 170 (%)	Period 7 19 Sep–11 Oct n = 200 (%)	Period 8 15 Dec–13 Jan n = 200 (%)	p value
SARS-CoV-2 RT-PCR					
Infection rate	15 (7.7)	6 (3.5)	33 (16.7)	16 (8.0)	** < 0.001**
95% confidence interval	4.4 to 12.4	1.3 to 7.5	11.6 to 22.4	4.6 to 12.7	
SARS-CoV-2 antibodies*					
Seroprevalence	182 (97.8)	167 (98.2)	191 (97.9)	199 (99.5)	0.536
95% confidence interval	94.6 to 99.4	94.9 to 99.6	94.8 to 99.4	97.2 to 99.9	
Geometric mean concentration (SD)**	128 (2.7)	54.2 (3.0)	57.9 (3.5)	66.9 (2.9)	** < 0.001**

^*^Invalid specimen, n (%): period 5–9 (4.6), period 7–5 (2.5)

^**^Geometric mean concentrations were calculated among seropositive only

**Fig. 4:**
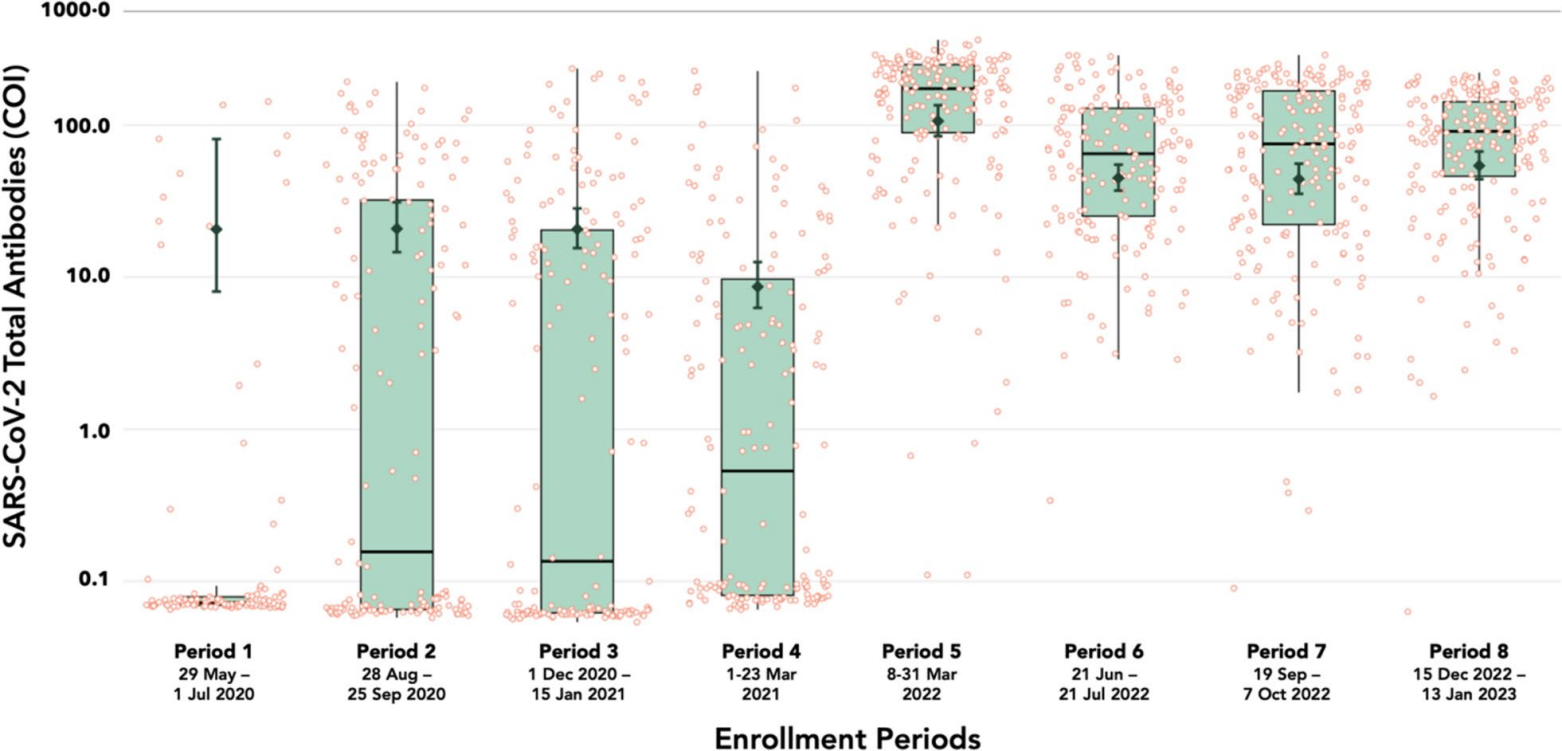
Comparison of SARS-CoV-2 seroprevalence in Manila across all enrollment periods. Green boxplots indicate the distribution of antibody levels; red points indicate individual data points; dark green error bars indicate the geometric mean concentration among seropositives. *COI* cutoff index

The seroprevalence in periods 5–8 were significantly greater compared to the seroprevalence across periods 1–4 which were measured before the implementation of the national vaccination program, as previously reported [12] (Fig. 4).

We initially intended to compare seropositive GMC estimates by vaccination status and type of vaccine brand received as primary series, stratified by period of data collection; however, due to the small size of participants belonging to strata of vaccination status, our estimates would have been unreliable and difficult to interpret.

### Infection rate

**Fig. 5:**
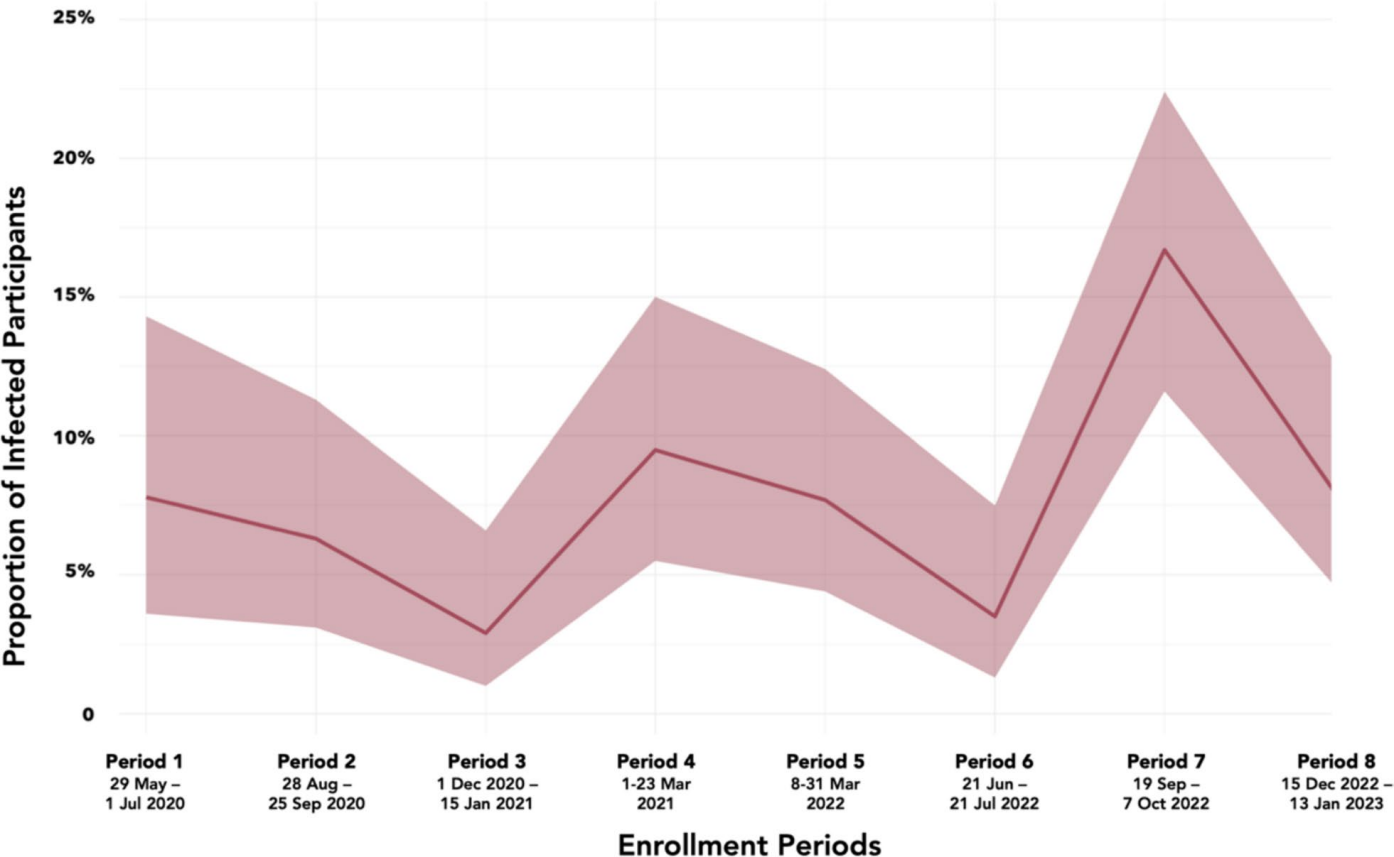
Comparison of SARS-CoV-2 infection rate in Manila across all enrollment periods

Across Periods 5–8, infection rates were comparable at < 10%, except for Period 7 (16.7%), which follows a relatively small rise and fall in reported new cases in Metro Manila in the preceding 3 months and coincides with another rise in reported cases (Table 3, Fig. 1). Except for Period 7, infection rates were also comparable across all other data collection periods (Fig. 5).

## Discussion

We aimed to describe SARS-CoV-2 seroprevalence among outpatient attendees in a non-respiratory, non-fever clinic in Manila a year after the commencement of the national COVID-19 immunization program and found population-level seroprevalence to be consistently high (97.8–99.5%) on repeated measurements across four periods, 3 months apart. Among seropositive individuals, the GMC of SARS-CoV-2 antibodies were significantly different across periods, roughly reflecting the changing COVID-19 epidemiology in the National Capital Region and suggesting some degree of waning as early as 3 months. The observed decline in GMC among seropositive individuals between periods, especially in the background of relatively stable high seroprevalence overall, could also be explained by the relatively more rapid waning of anti-N antibodies compared to other antibodies such as anti-spike and anti-SARS-CoV-2 receptor binding domain [23, 24]. By estimating seroprevalence rates before and after vaccine program implementation in the same catchment area, our study adds richness to the seroepidemiological description of community dwellers in one of the cities in the world most heavily affected by the pandemic. The consistently high seroprevalence rates we observed after immunization were comparable to estimates seen in repeated assessments from larger population-based studies that did not differentiate between immunization- and infection-induced seropositivity [6, 25, 26].

In the same population, we found infection rates to be comparable across different periods except for one sampling that took place immediately after a rise in reported cases in the region. The low infection rates we observed were consistent with our expectations that symptomatic patients would not seek care in a non-respiratory, non-fever outpatient clinic; however, this finding also reiterates the potential extent of asymptomatic infections in the community.

The stark contrast between the consistently high seroprevalence and low infection rate and self-reported infection history in our study demonstrates the advantage of serological assessments in capturing population-level infection burden over time over symptom- and/or PCR-based surveillance strategies. Unlike PCR, serological tests can identify individuals previously infected by SARS-CoV-2 even if they never underwent testing while acutely ill [27], which is particularly relevant given that asymptomatic infections comprise approximately 40% of individuals with confirmed COVID-19 [28]. Our findings are consistent with other observations where true case counts using seroprevalence studies were estimated to be up to 9.3 times greater than reported case counts, highlighting how serosurveillance can improve the characterization of pandemic impact [29].

We also comprehensively described the COVID-19 vaccination history among our study participants. In line with the national vaccination program, the majority of those who completed the primary vaccine series received CoronaVac while majority of those who received any booster dose had Comirnaty. Almost all the participants sourced their vaccines through the local government units and government health facilities, consistent with the implementing guidelines. However, that a few study participants received vaccines through private companies hinted at the conflict between privatized schemes and government procurement at the time of scarcity early in the pandemic [30, 31]. While later it was clarified that private companies could legally procure their own vaccines for their employees, the national COVID-19 taskforce required private entities to share their doses for public use, dissuading many, while at the same time there was anecdotal evidence that vaccines were given to yet ineligible populations. We found a higher vaccination rate in the study (71.8–88.5%) compared to the national eligible population (53.1–65.2%) in the same period. This may be explained by better health-seeking behavior in the study population and by greater vaccine access among those living in Metro Manila compared to the rest of the country. We could not find reliable granular vaccine coverage estimates during the period, limiting our comparisons. Lastly, that < 10% of participants received influenza vaccine in the past year and < 5% received pneumococcal vaccine in the past 5 years allude to the existing health systems challenges in the country even before the pandemic and reiterate the need to coordinate efforts and improve vaccine accessibility across different diseases as part of preparation for the next pandemic.

Our study has several limitations which we have previously described in depth [12]. Briefly, the relatively small sample size across periods limits our ability to perform subgroup analyses, especially in comparing seroprevalence and antibody concentrations according to vaccine status and vaccine brands. However, our estimates of seroprevalence are adequately powered. An important limitation of our study is the use of an assay detecting anti-N antibody as an estimate of seroprevalence in a population where a substantial proportion (> 40%) received whole virion inactivated vaccine (i.e., Coronavac), which is known to induce this antibody response. While anti-N antibody levels following vaccination alone are typically lower and less durable than following natural infection [32, 33], this could still potentially contribute to the high seroprevalence observed and prevent differentiation between infection-induced and vaccination-induced immunity. Resource constraints precluded our capacity to measure neutralizing and other antibodies, making the ascertainment of population-level immunity difficult. Our study population also consisted of ABC attendees, which may differ from the general population. Patients seeking post-exposure prophylaxis for rabies may represent a more health-conscious population or those engaged in activities that could differentially impact COVID-19 exposure risk. Nevertheless, the high proportion of participants belonging to the 18–39 age group and low income households and having at least secondary education level mirrors the demographic and socioeconomic status of Metro Manila residents [34]. Despite these limitations, our analyses provide insights into the hybrid immunity following the national COVID-19 vaccination program and Omicron-driven wave among community dwellers in Manila and highlight the need to incorporate serosurveillance studies into future pandemic preparedness efforts.

## Conclusion

A year after implementation of the national COVID-19 vaccination program in the Philippines, the population-level seroprevalence among clinic attendees in Manila remained consistently high across different sampling periods, but antibody concentration analysis showed potential waning within a 3-month period. Differences in infection rates across sampling periods may reflect epidemiological waves in the community.

## Data Availability

No datasets were generated or analysed during the current study.
